# Artificial intelligence in pulmonary hypertension: a systematic review

**DOI:** 10.1186/s40001-025-03557-5

**Published:** 2025-12-08

**Authors:** Tilmann Kramer, Mira Kramer, Christian Hagist, Stefan Spinler

**Affiliations:** 1 Department of Internal Medicine III, Heart Center at the University of Cologne, Cologne, Germany; 2 Chair of Logistics Management (Kühne Foundation Endowed Chair), WHU - Otto Beisheim School of Management, Vallendar, Germany; 3 Department of Anesthesiology and Intensive Care Medicine, University Hospital of Ulm, Ulm, Germany; 4 Chair of Economic and Social Policy, WHU - Otto Beisheim School of Management, Vallendar, Germany

**Keywords:** Pulmonary hypertension, Pulmonary arterial hypertension, Artificial intelligence, Machine learning, Deep learning, Diagnostic and prognostic prediction models

## Abstract

**Background:**

Pulmonary hypertension (PH) is characterized by elevated pulmonary pressures and right ventricular strain. Pulmonary arterial hypertension (PAH), a subtype, has a poor prognosis, especially when diagnosis is delayed. Artificial intelligence (AI) methods, including machine learning (ML) and deep learning (DL), offer potential for non-invasive prediction and risk stratification.

**Objective:**

This systematic review assesses ML and DL applications for non-invasive diagnosis, classification, and prognostication in PH and PAH, with emphasis on methodological quality and clinical applicability.

**Methods:**

A PRISMA-guided search identified studies using ML or DL on non-invasive clinical, imaging, or biomarker data, including omics and laboratory parameters. Study characteristics and heterogeneity were synthesized using the SWiM framework. Risk of bias was assessed using PROBAST+AI across participant selection, predictors, outcomes, and analysis.

**Results:**

Fifty-three studies were included. Most used clinical, echocardiographic, imaging, or molecular data. AUC values ranged from 0.71 to 1.00. DL approaches, especially convolutional neural networks, were increasingly applied but seldom externally validated. Nine studies were multicenter, four prospective, one combined retrospective and prospective cohorts, none were randomized controlled trials. The rest were retrospective single-center studies. In 15 studies, right heart catheterization was either not performed or not clearly reported. SWiM analysis showed substantial heterogeneity in study design and outcome definitions. According to PROBAST +AI, 44 studies (83%) had low risk of bias, though applicability concerns were common.

**Conclusion:**

ML and DL models show promise for PH and PAH diagnosis and prognosis, but limitations in subclass differentiation, methodological transparency, and validation must be addressed in future research.

**Supplementary Information:**

The online version contains supplementary material available at 10.1186/s40001-025-03557-5.

## Introduction

Pulmonary hypertension (PH) is a progressive, life-limiting condition defined by a mean pulmonary arterial pressure (mPAP) above 20 mmHg at rest, as confirmed by right heart catheterization (RHC) [1]. PH encompasses a spectrum of entities with distinct etiologies, pathophysiologies, and therapeutic implications. The international classification system endorsed by the World Symposium on PH and reaffirmed in the 2022 ESC/ERS guidelines subdivides PH into five groups, including pulmonary arterial hypertension (PAH), PH due to left heart or lung disease, chronic thromboembolic PH (CTEPH), and multifactorial forms [1]. Among these, PAH is a rare but severe vascular disorder characterized by progressive remodeling of the pulmonary arteries and increased pulmonary vascular resistance, often leading to right heart failure and systemic complications [1, 2].

Despite advances in targeted pharmacologic therapy and structured follow-up, survival remains limited, and many patients present at an advanced stage due to significant diagnostic delays [3–6]. These delays, largely attributable to non-specific symptoms such as dyspnea, fatigue, and reduced exercise tolerance [1, 5] average approximately 2.5 years and are associated with increased mortality and higher healthcare utilization [6].

Echocardiography remains the first-line screening tool, though its interpretation is prone to interobserver variability [1, 7], whereas RHC provides definitive diagnosis but is invasive and less feasible for large-scale screening [1, 8].

In this context, artificial intelligence (AI) has emerged as a promising tool to enhance early detection, improve risk stratification, and support clinical decision-making in PH [9–11]. Machine learning (ML) and deep learning (DL) algorithms can identify complex, nonlinear relationships within high-dimensional datasets, enabling earlier and more accurate recognition of disease patterns [9, 12, 13]. Recent studies have applied AI to detect PH using chest radiographs, electrocardiograms, and multimodal combinations of clinical and diagnostic data, with performance in some cases comparable to or exceeding that of physicians [14–17]. ML models trained on real-world electronic health records (EHRs) have shown potential to identify at-risk patients before clinical diagnosis, possibly helping to reduce diagnostic delay [18].

Beyond detection, AI has been used to predict disease severity, treatment response, and clinical outcomes based on diverse data inputs, including clinical parameters, imaging results, laboratory values, and omics data, often relying on multimodal architectures that approximate complex clinical reasoning [12, 16, 19].

However, existing studies vary considerably in methodological quality and clinical applicability [9, 12], and it remains uncertain whether models trained on mixed PH populations can generalize across subtypes [12, 20].This systematic review synthesizes the current evidence on ML and DL applications in PH, focusing on modeled PH subtypes, data modalities, algorithmic techniques, reported outcomes, and methodological rigor, including validation strategies and overfitting control. Given that nuanced diagnostic distinctions between PH subtypes carry significant therapeutic implications, precise attribution is essential in AI research on PH to ensure clinically meaningful translation [1]. This review aims to provide an evidence-based overview and to guide future research at the intersection of PH phenotyping and advanced AI methodologies.

## Methods

This systematic review was conducted in accordance with the Preferred Reporting Items for Systematic Reviews and Meta-Analyses (PRISMA) 2020 guidelines [21] and was prospectively registered in the International Prospective Register of Systematic Reviews (PROSPERO; registration number: CRD420251074202) [22]. The review focused on ML and DL applications in PH, emphasizing diagnostic, phenotypic, and prognostic use cases. Methodological aspects such as data types, algorithmic approaches, validation strategies, and subtype attribution were systematically assessed.

A comprehensive literature search was carried out using two databases: MEDLINE via PubMed and Google Scholar. Google Scholar was searched to identify additional relevant studies. The complete search strategy, including specific Medical Subject Headings (MeSH) terms, free-text keywords, Boolean operators, and field specifications, is provided in Supplementary Table S1. The strategy was designed to identify peer-reviewed original research articles applying AI methods in PH. MeSH terms and free-text keywords such as “pulmonary hypertension”, “pulmonary arterial hypertension”, “machine learning”, “deep learning”, “artificial intelligence”, “diagnosis”, “phenotyping”, “prognosis”, “prediction”, “non-invasive”, “risk stratification”, “survival”, “mortality”, “electrocardiography”, “echocardiography”, “chest X-ray”, “computed tomography”, “magnetic resonance imaging”, and “electronic health records” were used. Boolean operators (“AND”, “OR”) were applied to combine related terms, and searches were conducted within titles, abstracts, and MeSH terms. The same search strategy was applied across both databases, with minor syntax adjustments for Google Scholar, which also indexes full-text information. The search was restricted to English-language publications published since 2016 to reflect the emergence of modern ML and DL approaches in PH research. Only studies involving human subjects were included. In addition to database searches, reference lists of the electronically identified articles were screened manually, yielding six additional studies. The strategy was further refined through iterative testing to ensure retrieval of all known eligible studies. Refinement involved adjusting keyword groupings and Boolean logic to verify that previously known relevant publications were consistently retrieved by the final search strategy.

Studies were included if they met the predefined eligibility criteria. Specifically, studies were eligible if they (i) applied ML or DL techniques to predict or classify PH; (ii) used non-invasive data as input features, such as clinical parameters, laboratory results, electrocardiographic measurements, echocardiography, chest imaging, or other routinely collected non-invasive modalities; (iii) reported quantitative predictive performance metrics for diagnostic, classification, or prognostic tasks; and (iv) provided sufficient methodological detail to allow an assessment of model development, validation, and reproducibility.

Studies were excluded if they (i) were reviews, editorials, conference abstracts, or case reports; (ii) exclusively relied on invasive input features, such as hemodynamic parameters from RHC; or (iii) did not report relevant outcome metrics for model performance.

Two reviewers independently screened titles and abstracts for eligibility. Full-text articles of potentially eligible studies were then reviewed in detail. Disagreements were resolved by discussion and, if necessary, by consulting a third reviewer. The same two reviewers independently extracted data from each included study using a pre-defined data extraction form. Extracted information comprised authorship, year of publication, study location, population characteristics, PH subgroup investigated, data type and source, model type and structure, clinical objective (diagnostic classification, phenotypic differentiation, or prognostic prediction), model performance metrics including area under the receiver operating characteristic curve (AUC), and methods used for validation.

To systematically assess the risk of bias, methodological quality, and applicability of model development and evaluation, the updated Prediction Model Risk of Bias Assessment Tool for Artificial Intelligence (PROBAST+AI) was applied to all included studies [23]. This tool is applicable to AI-based prediction models and evaluates the risk of bias across four domains: participants, predictors, outcome, and analysis. It includes additional signaling questions tailored to ML workflows, addressing aspects such as model calibration, resampling methods, data leakage, and model explainability. To address domain-specific concerns in PH, the assessment was extended to evaluate whether studies applied guideline-based hemodynamic criteria for cohort labeling and adhered to consistent subtype classification according to current recommendations [1]. This approach ensured a robust appraisal of AI model quality in relation to the underlying ground truth and the respective PH populations.

All steps of study selection, data extraction, and quality appraisal were conducted in accordance with established best practices for systematic reviews of prediction model studies. Due to substantial methodological heterogeneity in model types, input features, and clinical endpoints, no meta-analysis was performed. Instead, findings were synthesized systematically. Studies were grouped according to clinical objective, algorithmic approach, outcome parameters, performance metrics, validation strategy, and PH subtype, wherever classification was possible based on the reported data and in accordance with the current ESC/ERS guidelines and Nice classification [1]. This synthesis approach followed the Synthesis Without Meta-analysis (SWiM) guideline to ensure transparent and structured reporting in the absence of a meta-analysis [24]. Study characteristics were extracted and systematically tabulated, incorporating relevant clinical and modeling features.

## Results

### Study selection and characteristics

**Fig. 1:**
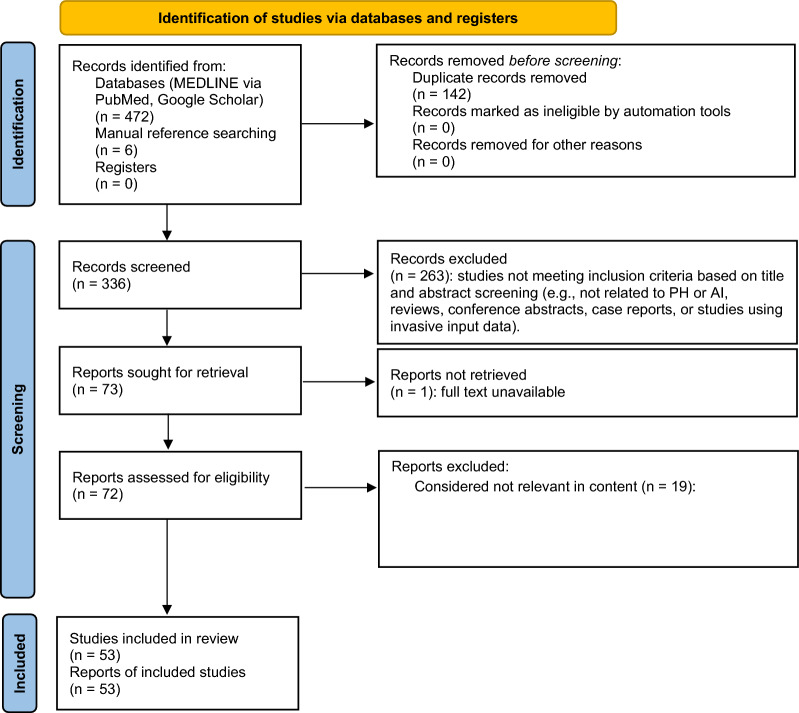
PRISMA flowchart for study selection. This flowchart illustrates the study selection process in accordance with the PRISMA 2020 guidelines [21]. A total of 472 records were identified through database searches (MEDLINE via PubMed and Google Scholar), and an additional six studies were identified through manual reference searching of the previously selected studies. After removing 142 duplicates, 336 records remained for title and abstract screening. Of these, 263 were excluded based on predefined eligibility criteria. Seventy-two full-text articles were assessed for eligibility, of which 19 articles were excluded. Ultimately, 53 studies were included in the final qualitative synthesis. Reasons for exclusion at each stage are detailed in the flowchart

A total of 53 studies met the predefined eligibility criteria and were included in this systematic review. The study selection process is illustrated in the PRISMA flow chart (Fig. 1). The corresponding PRISMA 2020 Checklist detailing adherence to reporting standards is provided in Supplementary Table S2.

Results were synthesized narratively and organized by clinical objective, algorithmic approach and outcome parameters in line with SWiM guidance. Study-level characteristics such as study design, sample size, PH classification, input data types, clinical objectives, validation strategies, model types, and performance metrics are systematically presented in Table 1. The table also summarizes outcome definitions, use of prognostic modeling and key strengths and limitations for each study. Risk of bias and applicability concerns were assessed using PROBAST-AI and are detailed in Supplementary Table S3, structured by predefined domains.

**Table 1:** Study characteristics and performance metrics

Author (year)	PH group	Study group	Study design	Sample size	Key findings	Outcome measures	Prognostic model used	Model type	Diagnosis method	Validation method	Strengths and limitations
Alabed et al. (2022) [25]	PAH	MPCA-based ML for Mortality Prediction in PAH	Retro-spective cohort	723 patients	MPCA-based features from CMR significantly improved 1-year mortality prediction (c-index: 0.83 vs. 0.71 with REVEAL)	c-index, ROC-AUC, Kaplan–Meier survival	MPCA, CMR features, REVEAL score	ML	RHC	Tenfold Cross-validation, Internal validation	Strengths: Transparent, clinically interpretable; Limitations: Retrospective, single center, no external validation
Anand et al. (2024) [26]	PH	ML for PH Diagnosis Using Echo	Retro-spective cohort	7853 patients	XGBoost model for PH detection achieved high AUC (0.83) and sensitivity (88%) with specificity (54%)	AUC, Accuracy, Sensitivity, Specificity, PPV, NPV	XGBoost	ML	RHC	Fivefold Cross-validation, Internal validation	Strengths: Large cohort, no need for TR jet velocity; Limitations: Retrospective, High PH prevalence in cohort, model performance drop in testing data
Aras et al. (2023) [27]	PAH, PH	DL ECG Detection of PH	Retro-spective cohort	24,470 patients	CNN model achieved high AUC for detecting PH (AUC: 0.89), sensitivity (0.79), and specificity (0.84). For pre-capillary PH, the model performed excellently (AUC: 0.91). For PAH, AUC was 0.88	AUC, Sensitivity, Specificity, PPV, NPV, F1-Score	CNN	DL	RHC or Echo, RHC (subgroup)	Internal validation	Strengths: Large cohort, early detection capability (up to 2 years before diagnosis), potential for widespread clinical use with remote monitoring; Limitations: Retrospective design, misclassification potential for some PH subtypes due to broad inclusion criterion (TR-Velocity > 3.4 m/s), dependent on quality of ECG data
Argiento et al. (2024) [28]	PAH	ML for PAH Prediction	Retro-spective cohort	226 patients	Developed an ML algorithm for identifying PAH from anamnesis and non-invasive data. AUC of 83%, accuracy of 74%	AUC, Sensitivity, Specificity	Elastic-Net Regularized Generalized Linear Model	ML	RHC	Threefold Cross-validation, Internal validation	Strengths: Focus on high-risk populations, robust ML model. Limitations: Retrospective, Single center, unbalanced sample, smaller dataset
Bauer et al. (2021) [29]	PAH	ML for PAH Prediction Using Proteomics Data	Retro-spective cohort	157	ML models using proteomics data showed significant potential in predicting PAH with high AUC, sensitivity, and specificity, outperforming traditional biomarkers	AUC, Sensitivity, Specificity	Random Forest	ML	RHC	Tenfold Cross-validation, Internal validation	Strengths: Use of proteomics for early detection, potential for personalized treatment; Limitations: Retrospective, sample size, and lack of external validation
Bordag et al. (2023) [30]	PAH, PH (left heart disease), PH (lung disease), CTEPH	ML for PH Prediction Using Lipidomics	Retro-spective cohort	233 patients	ML models using lipidomics identified diagnostic and prognostic biomarkers, with predictive potential (AUC 0.82–0.90) for PH	AUC, Sensitivity, Specificity	Random Forest, XGBoost	ML	RHC	Sevenfold cross-validation, External Validation	Strengths: Novel lipidomics approach, high diagnostic accuracy; Limitations: Small sample size, single center, potential bias in prognostic scores with mixed PH groups
Chettrit et al. (2019) [31]	COPD-related PH	DL System for PH Risk Stratification using chest CT	Retro-spective cohort	1285 chest CT studies	The DL model automated the measurement of pulmonary artery (PA) and aorta (Ao) diameters to assess the PA-to-Ao ratio, showing significant potential for PH risk stratification with high Pearson correlation (93% for Ao, 92% for PA)	Pearson correlation, Sensitivity, Specificity, PPV	CNN	DL	Diagnosis based on clinical criteria (RHC, Echo, and other diagnostic tests)	Cross-validation, Internal validation	Strengths: Fully automated, accurate measurements, high specificity for screening; Limitations: Retrospective design, reliance on contrast-enhanced CT scans, potential bias with mixed PH groups
Dawes et al. (2017) [66]	PH	ML of 3D Right Ventricular Motion for survival prediction in PH	Prospective cohort	256 patients	Survival prediction improved with 3D right ventricular motion data. Model provided better prediction than conventional clinical measures (AUC: 0.73 vs. 0.60, *P* < 0.001)	AUC, Sensitivity, Survival Time	Principal Component Analysis, Supervised Learning	ML	RHC	Eightfold Cross-validation	Strengths: Incorporates 3D motion, better survival prediction, prospective design; Limitations: Retrospective data analysis
Diller et al. (2022) [32]	PAH	DL Framework for Detection and Prognostication of PAH	Retro-spective cohort	450 patients	DL model achieved 97.6% accuracy and 100% sensitivity in detecting PAH. It also provided prognostic insights with non-inferior performance compared to expert echocardiography	Sensitivity, Specificity, AUC, Cox-proportional hazard models	CNN-based segmentation and feature extraction; prognostic modelling via multivariable Cox regression	DL	RHC	Internal validation	Strengths: High accuracy, expert-level prediction, provides prognostic data; Limitations: Limited to expert center data, small number of normal controls, retrospective design
DuBrock et al. (2024) [33]	PAH	DL algorithm for early detection of PH based on 12-lead ECG	Retro-spective cohort	39,823 PH-likely patients, 219,404 controls	DL model achieved high accuracy for detecting PH, with an AUC of 0.92 at Mayo Clinic, and 0.88 at VUMC. The model was capable of predicting PH up to 5 years prior to diagnosis	AUC, Sensitivity, Specificity, PPV, NPV	CNN	DL	RHC or Echo	Internal validation, External validation (VUMC)	Strengths: High performance, early detection capability; Limitations: Retrospective design, reliance on RHC and TRV measurements, potential bias due to using echo and RHC for cohort definition
Duo et al. (2022) [34]	PAH	Gene expression-based diagnostic signature for PAH	Retro-spective cohort	73 PAH samples, 36 normal samples	A diagnostic signature (PDS) for PAH was constructed from key genes identified via WGCNA and LASSO. ROC analysis showed AUCs of 0.948 and 0.945 in two independent cohorts	Sensitivity, Specificity, AUC	LASSO	ML	RHC	External validation (GSE113439)	Strengths: High accuracy, identification of key biomarkers and immune landscape; Limitations: Limited sample size, experimental validation needed
Dwivedi et al. (2024) [35]	PAH and PH-LD	AI model for lung fibrosis quantification and survival prediction	Retro-spective cohort	521 patients	AI-quantified lung fibrosis on CT pulmonary angiograms was associated with increased mortality risk (C-index: 0.76). Combining AI with radiologic scoring improved survival prediction	C-index, Mortality	AI-based DL Model	DL	RHC	External validation	Strengths: AI model for accurate fibrosis quantification; Limitations: Retrospective study, reliance on external validation, potential bias from image acquisition variability
Errington et al. (2023) [36]	PAH	miRNA expression-based ML model for PAH diagnosis	Retro-spective cohort	107 patients	ML model based on miRNA expression showed high diagnostic accuracy (AUC: 0.85) for PAH and provided potential biomarkers for prognosis	AUC, Sensitivity, Specificity, PPV, NPV	SVM, Random Forest, LASSO, XGBoost, Ensemble, Rpart	ML	RHC	Tenfold Cross-validation, External validation	Strengths: High diagnostic accuracy, identification of miRNA biomarkers for PAH; Limitations: Limited external validation, retrospective design
Fortmeier et al. (2022) [37]	PH	XGBoost model for mPAP prediction	Retro-spective cohort	116 patients	XGBoost model based on echocardiographic parameters was able to predict mPAP and associated with 2-year all-cause mortality (HR 2.4)	Pearson correlation, survival	XGBoost	ML	RHC	Internal and external validation	Strengths: Cohort with both RHC and echo data; Limitations: Small sample size, retrospective design
Gawlitza et al. (2024) [38]	CTEPH	ML-based feature identification for hemodynamic endpoint prediction using CT	Retro-spective cohort	127 patients	The random forest model achieved AUC of 0.82 for mPAP prediction and 0.74 for PA SaO2 prediction, using quantitative and qualitative CT features	AUC, Sensitivity, Specificity, PPV, NPV	Random Forest	ML	RHC	Cross-validation, internal validation	Strengths: Non-invasive risk stratification using CT features; Limitations: Small cohort, Retrospective design
Imai et al. (2024) [14]	PAH	DL algorithm for PAH detection using CXR	Retro-spective cohort	145 PAH patients, 260 controls	The DL model (ResNet50) achieved AUC of 0.988 for PAH detection using CXR images, outperforming experienced doctors (AUC 0.945)	AUC, Sensitivity, Specificity	ResNet50	DL	RHC	Fourfold cross-validation, Internal validation	Strengths: High diagnostic accuracy, non-invasive and cost-effective; Limitations: Small sample size, single center study, potential image quality variability, retrospective design
Kanwar et al. (2020) [41]	PAH	Bayesian network (PHORA) for PAH risk stratification	Retro-spective cohort	3515 patients from the REVEAL registry	The PHORA Bayesian network model achieved AUC of 0.80 for 1-year survival, outperforming the REVEAL 2.0 model (AUC 0.76). It was validated externally in two registries with an AUC of 0.74 and 0.80	AUC, Sensitivity, Specificity, NPV, PPV	Bayesian network (Tree-augmented Naïve Bayes—TAN)	ML	RHC	Internal validation (REVEAL registry), External validation (COMPERA, PHSANZ)	Strengths: Improved discriminatory ability, can handle missing data, validated in multiple cohorts; Limitations: Survival bias, missing data in registries
Kheyfets et al. (2023) [68]	PAH	Random forest model for PAH survival prediction using clinical and biomarker data	Prospective cohort	167 PAH patients	The random forest model predicted 4-year survival risk with AUC 0.94 (internal validation) and AUC 0.81 (external validation). It identified novel biomarkers such as IL-2, IL-9, and 6MWD as significant predictors of risk	AUC, Sensitivity, Specificity	Random Forest	ML	RHC	Internal validation (Stanford cohort), External validation (Sheffield cohort)	Strengths: Novel approach combining clinical and biomarker data for personalized PAH prognostication, prospective design; Limitations: Relatively small cohort, biomarker data from a single center
Kiely et al. (2019) [42]	iPAH	Predictive model based on HCRU to identify patients at risk for iPAH	Retro-spective cohort	709 iPAH patients and 2,812,458 non-iPAH patients	The Gradient Boosting Trees model achieved 99.99% specificity and 14.10% sensitivity, identifying 100 iPAH cases among 969 flagged patients	Sensitivity, Specificity, PPV, NPV	Gradient Boosting Trees (XGBoost)	ML	RHC	Fivefold cross-validation, internal validation	Strengths: Cost-effective, real-world data-based model for rare disease screening; Limitations: Low sensitivity, narrow scope of healthcare data used
Kogan et al. (2023) [18]	PAH, CTEPH, and other PH types	XGBoost model for early PH detection using EHR data	Retro-spective cohort	115,822 patients, 11,279,478 controls	The XGBoost model achieved AUC 0.92 for PH prediction. The model also predicted PH subgroups (PAH: 0.79–0.90 AUC, CTEPH: 0.87–0.96 AUC)	AUC, Sensitivity, PPV	XGBoost	ML	Echo or RHC	Threefold cross-validation, internal validation	Strengths: Large cohort, uses real-world EHR data; Limitations: PH diagnosis not uniformly confirmed by RHC, potential bias from coding algorithms, retrospective design
Kusunose et al. (2020) [45]	PH	DL model for PH detection using CXR	Retro-spective cohort	900 patients	CNN achieved AUC of 0.71 for PH detection using CXR images, improving significantly compared to human observers	AUC, NPV	CNN	DL	RHC	Tenfold cross-validation, internal and external validation	Strengths: AI-driven approach with CXR, non-invasive screening; Limitations: Moderate accuracy, single-center
Leha et al. (2019) [47]	PAH, PH due to left heart disease, PH due to lung disease and hypoxia, PH with unclear and multi-factorial	ML for PH prediction using echo	Retro-spective cohort	90 patients (68 with confirmed PH, 22 without PH)	AUC for SVM: 0.83, Random Forest Regression: 0.87 for predicting PH from echo	AUC, Sensitivity, Specificity, PPV, NPV	SVM, Random Forest, Lasso, boosted classification trees	ML	RHC	Threefold cross-validation, Internal validation	Strengths: High AUC, broad echocardiographic data; Limitations: Small cohort, retrospective design
Liao et al. (2023) [69]	PH due to left heart disease, PAH, CTED	ML for PH detection using echo	Retro-spective cohort	346 patients	The ML model achieved AUC 0.945 in internal validation and AUC 0.950 in external validation for predicting PH from echocardiographic images	AUC	Linear regression,LightGBM, CatBoost	ML	RHC	Cross-validation (50%), Internal and external validation	Strengths: High AUC, robust model for PH detection from echocardiographic images, external validation; Limitations: relatively small sample size, no data on ethnic diversity, Possible bias in image quality, retrospective design
Lungu et al. (2016) [49]	PAH, PH due to left heart disease, PH due to lung disease and hypoxia, CTEPH, PH with unclear and multi-factorial	MRI-based ML model for PH detection	Retro-spective cohort	72 patients	The ML model achieved 92% accuracy in diagnosing PH using MRI-derived parameters and decision tree analysis	AUC, Sensitivity, Specificity, PPV, NPV	Random Forest Classification	ML	RHC	Leave-one-out cross-validation	Strengths: Non-invasive diagnostic tool, high accuracy with MRI; Limitations: Small sample size, lack of internal or external validation dataset, retrospective design, single center
Matsunaga et al. (2024) [50]	CTEPH	ML models for predicting mPAP in CTEPH	Retro-spective cohort	136 patients	The linear regression model achieved the highest R^2^ value of 0.388. Models including age, BNP, TRPG, CXR performed better than traditional methods using TRPG alone	R^2^, RMSE, MAE	Linear regression, Decision Tree, SVR, KNN, Random Forest, XGBoost	ML	RHC	Internal validation	Strengths: Multiple model types tested, multivariable model increases prediction accuracy; Limitations: Small sample size, no external validation
Murayama et al. (2024) [51]	PAH, CTEPH	DL model for RVEF estimation using 2D echo	Retro-spective cohort	93 patients	The DL model (3D-ResNet50) predicted RVEF with a mean absolute error of 7.67% and showed AUC 0.84 for detecting severe RV dysfunction	AUC, Mean absolute error	3D-ResNet50 CNN model	DL	RHC	Fivefold cross-validation, Internal validation	Strengths: Automated tool for RVEF prediction, high diagnostic accuracy, Echo-based; Limitations: Small sample size, retrospective design, proportional error observed
Nemati et al. (2024) [20]	PH	ML model for PH detection using orthogonal voltage gradient (OVG) and photoplethysmographic (PPG) signals	Retro-spective cohort	488 patients	AUC of 0.93, sensitivity of 87%, specificity of 83% for PH detection using non-invasive sensors (OVG & PPG signals)	AUC, Sensitivity, Specificity	Elastic Net, Random Forest	ML	RHC	Out-of-fold cross-validation, Internal validation	Strengths: Non-invasive, point-of-care, high sensitivity and specificity, generalizable; Limitations: Relies on specific features, limited to point-of-care application, retrospective design, still requires further validation
Ong et al. (2020) [52]	PH, PAH	Claims-based ML model for PH detection using EHR and Medicare claims	Retro-spective cohort	550 patients	ML models outperformed rule-based algorithms for identifying PH in administrative claims, achieving an AUC of 0.88	AUC, Sensitivity, Specificity, PPV, NPV	Lasso, Random Forest, Gradient boosting machine	ML	RHC	Tenfold cross-validation, Internal validation, Bootstrap	Strengths: High performance, real-world healthcare data, multicenter design; Limitations: Relies on administrative claims, no full external validation, retrospective design
Priya et al. (2021) [53]	PAH, PH due to left heart disease, PH due to lung disease and hypoxia, CTEPH, PH with unclear and multi-factorial	Cardiac MRI-based radiomics for PH detection using texture features	Retro-spective cohort	72 patients (42 PH, 30 controls)	The radiomics-based model achieved AUC 0.862 for PH detection and AUC 0.918 for PH patients with preserved LVEF in subgroup analysis	AUC, Sensitivity, Specificity, Accuracy	MLP, Random Forest, SVM, Elastic Net, Ridge	ML	RHC	Five-fold cross-validation	Strengths: Non-invasive, good diagnostic performance; Limitations: Small sample size, no external validation, single institution retrospective design
Priya et al. (2021) [54]	PAH, PH due to left heart disease, PH due to lung disease and hypoxia, CTEPH, PH with unclear and multi-factorial	Cardiac MRI-derived radiomics with DAFIT for PH detection	Retro-spective cohort	82 patients (42 PH, 40 controls)	DAFIT model with combined LV and RV masks performed with AUC 0.958, outperforming other models and showing superior predictive performance in PH detection	AUC	Linear, logistic, ridge, elastic net, and LASSO regression, Neural network, SVM, MLP, Random Forest, generalized boosted regression model	ML	RHC	Five-fold cross-validation	Strengths: High AUC, non-invasive, Data augmentation approach improves reproducibility; Limitations: Small sample size, Lack of external validation, limited PH subgroup, retrospective design
Schuler et al. (2022) [56]	PAH	ML model using ICD-9/10 codes, RHC, and PAH medication for PAH prediction	Retro-spective cohort	194 PAH patients and 786 controls	ML algorithm achieved sensitivity 0.88, specificity 0.93, PPV 0.89, NPV 0.92 in identifying PAH using administrative claims data	Sensitivity, Specificity, PPV, NPV, AUC	Random Forest, XGBoost, Elastic Net	ML	RHC	Tenfold cross-validation, Internal and external validation	Strengths: High sensitivity and specificity, External validation, non-invasive administrative data use; Limitations: Relies on administrative data, retrospective design
Shikhare et al. (2022) [57]	CTEPH	ML-based algorithm for right-to-left ventricle ratio (dRV/dLV) prediction from CTPA	Retro-spective cohort	125 patients	ML-based algorithm performed well with a strong correlation of r = 0.96 between predicted and manual dRV/dLV, associated with long ICU length of stay	AUC, Sensitivity, ICU length of stay	Neural Networks, CNN	ML	RHC	–	Strengths: High correlation with manual measurements, predictive of ICU length of stay, non-invasive; Limitations: 20% algorithm failure, small cohort, no validation on test set, single center, retrospective design
Suvon et al. (2023) [58]	PAH	Multimodal learning for mortality prediction using EHR, echo, and MRI data	Retro-spective cohort	2563 patients	The multimodal model combined numerical imaging features, categorical features, and textual features from EHR data, achieving AUC 0.89 for one-year mortality prediction	AUC, Sensitivity, Specificity, PPV, NPV	Bidirectional Encoder Representations from Transformers (BERT), MLP	ML	RHC	Tenfold cross-validation, Internal validation	Strengths: Multimodal approach, high AUC, utilizes real-world data; Limitations: Missing data, small class imbalance, retrospective design
Sweatt et al. (2019) [70]	PAH	Immune phenotypes classification using proteomic profiles	Prospective obser-vational	385 patients (discovery: 281, validation: 104)	Identified 4 immune clusters with distinct cytokine profiles using unsupervised ML, which correlated with clinical outcomes and 5-year survival	Survival Rate, Kaplan–Meier estimates, Cytokine levels	Consensus Clustering, Partial Correlation Networks	ML	RHC	External validation	Strengths: Unsupervised phenotyping, identifies immune phenotypes, links to prognosis, prospective multicenter design, external validation; Limitations: One-time point sampling, no dynamic monitoring
Swift et al. (2020) [59]	PAH	Tensor-based ML for CMR feature extraction to predict PAH	Retro-spective cohort	220 patients (150 with PAH, 70 with no PH)	Tensor-based ML approach showed AUC = 0.92 for PAH diagnosis using CMR data, identifying new diagnostic features	AUC, Sensitivity, Specificity, PPV, NPV	Tensor-based ML, Multilinear Subspace Learning (MPCA)	ML	RHC	Tenfold cross-validation	Strengths: High diagnostic accuracy, innovative approach using CMR data; Limitations: Small sample size, requires CMR, single center, no external validation retrospective design
Swinnen et al. (2023) [60]	PAH vs. PH due to left heart disease (PH-LHD)	Differentiation of PAH from PH-LHD using ML on noninvasive data	Retro-spective cohort	344 patients	Random Forest-based model showed sensitivity of 64% and 100% specificity for PH-LHD detection; outperforming the Jacobs score	AUC, Sensitivity, Specificity, PPV, NPV	Random Forest, Logistic Regression	ML	RHC	Tenfold cross-validation, Internal validation	Strengths: Highly specific model, non-invasive approach to differentiate PAH vs PH-LHD; Limitations: Retrospective design, single center
Zhang et al. (2023) [63]	PAH, PH due to left heart disease, PH due to lung disease and hypoxia, CTEPH, PH with unclear and multi-factorial	ML-based PAP prediction from CTPA	Retro-spective cohort	55 patients	Developed ML model using CTPA for the automatic evaluation of PAP. Achieved good consistency between predicted and manual measurements for mPAP, sPAP, dPAP	Intraclass correlation coefficient (ICC), AUC, mPAP, sPAP, dPAP, TPR	XGBoost, SVM, CatBoost	ML, DL	RHC	Tenfold cross-validation	Strengths: Accurate PAP prediction and segmentation via CTPA; Limitations: Small sample size, retrospective design
Zhao et al. (2025) [71]	PH (pre- and postcapillary)	Multimodal DL for PH detection from EHR, echo, and CXR	Prospective and retro-spective design	2451 patients	Developed MMF-PH model integrating CXR, ECG, echo, and clinical data; outperformed Echo in PH screening with higher specificity and NPV across datasets	Accuracy, Precision, Sensitivity, Specificity, NPV, F1, AUROC, AUPRC	Multimodal DL (MMF-PH)	DL	RHC	Internal and external validation	Strengths: Robust diagnostic accuracy, non-invasive PH screening, multicenter design, partially prospective design; Limitations: Small external validation group, PH subtypes were not comprehensively classified, overfitting potential

This table summarizes the characteristics of the studies included in the systematic review. The “Author (year)” column lists the lead author and the publication year of each study. The “PH group” column identifies the PH subtype studied. The “Study group” column provides a brief description of the study’s focus. “Study design” describes the methodology used in each study, whether retrospective or prospective. “Sample size” lists the number of participants included in each study. The “Key findings” column highlights the primary outcomes or findings. “Outcome measures” refers to the specific performance metrics used in each study. “Prognostic model used" details the type of model applied for prediction or prognosis. The “Model type” column specifies whether the model was ML or DL. “Diagnosis method” outlines the diagnostic methods used for PH in the study. “Validation method” describes the validation strategy used. Finally, the “Strengths and limitations” column provides insights into the strengths and weaknesses of each study

The majority of studies were retrospective (48 studies, 90.6%) [14–18, 20, 25–65] and single-center (44 studies, 83.0%) [14–16, 18, 20, 25–40, 42–45, 47, 49–51, 53–63, 66–69] in design. Nine studies (17.0%) were conducted across multiple centers [17, 41, 46, 48, 52, 64, 65, 70, 71], four (7.5%) were designed prospectively [66–68, 70], and one (1.9%) included both retrospective and prospective cohorts [71]. All included studies were published between 2016 and 2025. No randomized controlled trials were identified. Study populations included patients with either PAH or broader PH, with varying degrees of diagnostic certainty and subtype attribution (see Table 1 for full study-level details).

The primary clinical objectives varied across studies: 47 studies (88.7%) addressed diagnostic classification [14–18, 20, 26–56, 59, 61–65, 67, 69–71], nine studies (17.0%) aimed at prognostic prediction [25, 31, 32, 35, 41, 57, 58, 66, 68], and one study (1.9%) focused on phenotypic subgroup differentiation [60]. There was some overlap, as several studies pursued more than one objective. Data sources were heterogeneous and included clinical variables, echocardiographic data, electrocardiograms (ECG), chest imaging [(chest X-ray (CXR), computed tomography (CT), magnetic resonance imaging (MRI)], laboratory parameters, and omics-based inputs. The latter were employed in seven studies (13.2%), including four based on proteomic or transcriptomic data [29, 34, 36, 70], two on radiomic features [53, 54], and one on lipidomics [30] (see Table 1).

### Algorithmic approaches and input modalities

Among the 53 included studies, 32 (60.4%) employed ML models such as random forests, support vector machines or gradient boosting. DL models, particularly convolutional neural networks (CNNs), were applied in 18 studies (34.0%), mainly for image-based classification tasks involving CXR, CT scans, echocardiographic images, MRI, or ECG data. Three studies (5.7%) combined ML and DL methods [17, 63, 64]. An increasing number of studies adopted multimodal frameworks that integrated structured clinical data with unstructured sources such as imaging or free-text reports. Input features differed considerably between studies. While most studies relied on clinical, imaging, and echocardiographic data, seven studies (13.2%) incorporated ECG-derived parameters [16, 17, 27, 33, 46, 48, 71]. Eight studies (15.1%) used biomarker data [29, 34, 36, 39, 61, 62, 68, 70]. Data preprocessing strategies, feature selection methods, and hyperparameter tuning procedures were reported inconsistently (Tables 1, 2).

**Table 2:** Study characteristics and performance metrics—studies without explicit RHC confirmation for diagnosis

Author (year)	PH group	Study group	Study design	Sample size	Key findings	Outcome measures	Prognostic model used	Model type	Diagnosis method	Validation method	Strengths and limitations
Guo et al. (2025) [67]	PH	DL model based on phonocardiograms for PH screening	Prospective cohort study	985 patients	The model achieved an AUC of 0.79 for detecting elevated PASP ≥ 40 mm Hg, with sensitivity of 0.73 and specificity of 0.74. Performance was better when using a per-patient approach (AUC 0.82)	AUC, Sensitivity, Specificity, PPV, NPV	CNN	DL	Echo	Fivefold cross-validation, internal validation	Strengths: Non-invasive, low-cost screening tool for PH using a digital stethoscope, prospective design; Limitations: Echocardiographic PASP used as ground truth instead of RHC
Han et al. (2024) [15]	PAH-CHD (Pulmonary Arterial Hypertension in Congenital Heart Disease)	AI model based on chest radiographs (CXR) for PAH-CHD diagnosis	Retro-spective study	3255 radiographs	AI model achieved AUC 0.948 for CHD diagnosis and AUC 0.778 for PAH-CHD detection. With AI assistance, radiologists’ performance improved significantly for both diagnoses	AUC, Sensitivity, Specificity, Accuracy, F1 Score	ResNet18 (DL)	DL	Echo (CHD diagnosis), Clinical Reports	Fivefold cross-validation, Internal validation cohort	Strengths: Non-invasive, easy-to-perform CXR with AI assistance; Limitations: Single center, PAH diagnosed by echo, small sample for specific CHD types, retrospective design
Hu et al. (2023) [39]	PAH	ML-based biomarker identification for PAH	Retro-spective cohort	3 Lung tissue samples from PAH patients	Identification of gene biomarkers that reliably distinguished PAH from controls	AUC	Gradient boosting decision tree	ML	Bio-informatics analysis, Gene expression data from public datasets	Fivefold cross-validation, External dataset validation (GSE53408)	Strengths: Comprehensive bioinformatics approach and experimental validation. Limitations: Small sample size, potential overfitting due to limited data
Hyde et al. (2023) [40]	PAH	Claims-based ML algorithm for PAH identification	Retro-spective cohort	1339 PAH and 4222 non-PAH patients	The random forest model distinguished PAH from non-PAH patients with AUC of 0.84 for 6 months prior to diagnosis, showing promising early identification capability	AUC, Recall, Precision, Accuracy	Random Forest	ML	Claims data-based (ICD-10 codes for PAH or PH, outpatient claims)	Fivefold cross-validation, Internal validation	Strengths: Claims data-based approach for early PAH (or PH) identification, real-world evidence; Limitations: Potential biases in claims data (PAH vs PH), missing data
Kishikawa et al. (2025) [17]	PH	Ensemble learning model for PH detection using ECG, CXR, and BNP	Retro-spective cohort	71,826 ECG data points, 4718 CXR data points, 4718 BNP data points	AUC 0.872 for ensemble model; improves cardiologists’ detection accuracy for PH from 65 to 74% using ECG, CXR, and BNP data	AUC, Sensitivity, Specificity, Accuracy, PPV, NPV	Ensemble learning model	ML, DL	Echo	Internal validation	Strengths: Multimodal model, multicenter design, improves accuracy in detecting PH; Limitations: Only cardiologists tested; small cohort; unspecific patient population; only echocardiographic diagnosis without subtype classification, potentially limiting treatment decisions, retrospective design
Kivrak et al. (2023) [43]	PAH, PH due to left heart disease, PH due to lung disease and hypoxia, PH with unclear and multi-factorial mechanism, and non-PH	AI-based classification of PH using Chest X-ray images	Retro-spective cohort	6642 X-ray images from 2005 patients	The DL model (EfficientNetb0) achieved accuracy of 86.14%, AUC of 0.945 for PH detection	Accuracy, Recall, Precision, F1 Score, AUC	EfficientNetb0, SVM	DL	CXR and clinical findings	Internal validation	Strengths: High performance with CXR for PH classification; Limitations: Unbalanced dataset, retrospective design, black-box AI, no reliable PH diagnosis (no RHC)
Kusunose et al. (2022) [44]	Exercise-induced PH	DL model for PH detection using CXR	Retro-spective cohort	142 patients	The DL model achieved an AUC of 0.71 adding predictive value over clinical and echocardiographic parameters at rest, improving AUC from 0.65 to 0.74	AUC	DL (Capsule Network with residual blocks)	DL	AI model	Tenfold cross-validation	Strengths: Non-invasive detection of exercise-induced PH using CXR and AI; Limitations: Small cohort, no RHC for diagnosis, Black-box nature of the model, retrospective design
Kwon et al. (2020) [46]	PH	DL model for PH prediction using ECG	Retro-spective cohort	38,241 patients (including 4096 PH patients)	The AI algorithm achieved AUC of 0.859 (internal validation) and 0.902 (external validation)	AUC, Sensitivity, NPV, PPV	DL (ensemble neural network, CNN)	DL	Echo	Internal and external validation	Strengths: High accuracy using ECG data for PH detection, multicenter cohort, external validation; Limitations: No RHC for PH confirmation, potential bias from data imbalances
Liu et al. (2025) [48]	PH	DL model combining ECG and CXR for elevated PAP detection	Retro-spective cohort	85,193 patients from Hospital A, 16,736 patients from Hospital B	The DL model achieved AUC 0.8644 in internal validation and AUC 0.8734 in external validation for detecting elevated PAP using a combination of ECG and CXR. It also predicted future left ventricular dysfunction and cardiovascular mortality	AUC, Sensitivity, Specificity, PPV, NPV, Hazard Ratio	CNN, XGBoost	DL	Echo	Internal and external validation	Strengths: High diagnostic accuracy and NPV, integrates ECG and CXR for early PH detection, external validation, multicenter design; Limitations: No RHC confirmation, retrospective design
Liu et al. (2022) [16]	PH	AI model using ECG and Echo for PH detection	Retro-spective cohort	41,097 patients	The AI model achieved AUC 0.88 for elevated PAP detection and predicted cardiovascular mortality. It outperformed conventional ECG diagnosis by cardiologists	AUC, Sensitivity, Specificity, accuracy, Hazard Ratio	Neural network	DL	Echo	Tenfold cross-validation, internal and external validation	Strengths: Good diagnostic accuracy (AUC 0.88), robust prediction for cardiovascular mortality, validated externally, large sample size; Limitations: No RHC confirmation, retrospective design, possible biases due to cohort
Ragnarsdottir et al. (2024) [55]	PH in newborns	Echo-based multi-view DL for predicting and classifying PH	Retro-spective cohort	270 newborns	Explainable multi-view DL model for predicting and classifying PH severity with F1-score of 0.84 for severity and 0.92 for binary detection. Results demonstrated that multi-view and spatio-temporal analysis helped significantly improve prediction	F1-score, AUROC, accuracy, Recall, Precision	CNN	DL	Echo	Tenfold cross-validation, Internal validation	Strengths: First automated PH severity prediction in newborns using echo, explainable model, high performance metrics; Limitations: Data imbalance, limited to newborns, retrospective design
Yang et al. (2024) [61]	PAH and PH (unclear)	Gene expression data from 65 samples (41 PAH, 24 controls) from GEO datasets GSE113439 and GSE15197 were used for PAH prediction	Retro-spective study	274 (unclear)	Lasso combined with Linear Discriminant Analysis achieved the best feature selection performance (AUC = 0.741); the resulting diagnostic model based on selected hub genes reached an AUC of 0.87	AUC	113 ML algorithms	ML	unclear	Cross-validation	Strengths: High AUC, well-selected biomarkers; Limitations: Small sample size, unclear use of RHC for diagnosis in dataset, lack of diverse validation datasets, retrospective design, several methodological limitations
Zeng et al. (2021) [62]	PAH	Identification of biomarkers and immune infiltration analysis in IPAH using bioinformatics	Retro-spective cohort	74 patients	Identified HBB, RNASE2, S100A9, and IL1R2 as biomarkers with high diagnostic value (AUC = 1) for IPAH detection. Immune infiltration differences noted between IPAH and controls	AUC, Sensitivity, Specificity, ROC curve	SVM-recursive feature elimination, Lasso	ML	unclear	Tenfold cross-validation, External validation	Strengths: Accurate biomarkers, Immune infiltration analysis; Limitations: Small dataset, relies on bioinformatics datasets, no real-time monitoring, unclear use of RHC for diagnosis in dataset, retrospective design
Zhao et al. (2024) [64]	CTEPH	Automated CTEPH detection using non-contrasted CT scans	Retro-spective cohort	300 patients	Developed a cascaded network with multiple instance learning using non-contrast CT scans, achieving an AUC of 0.807 and sensitivity of 0.795 in detecting CTEPH	AUC, Sensitivity, Specificity, Accuracy	ResNet-18 CNN	ML, DL	CTEPH diagnosis based on MSKCC Q-SPECT/CT and Modified PIOPED II criteria	Fivefold cross-validation, External validation	Strengths: Non-invasive approach with no additional annotations required. High diagnostic accuracy, multicenter design. Limitations: External validation is limited as the second cohort included only healthy subjects
Zou et al. (2020) [65]	PH	DL-based PH detection and PASP prediction from CXR	Retro-spective cohort	762 patients	DL approach using frontal CXR to screen for PH with high AUC (0.970) on internal test, 0.967 on external test	AUC, Sensitivity, Specificity, PPV, NPV, MAE	InceptionV3, Xception, ResNet50	DL	Echo	Eightfold cross-validation, Internal and external validation	Strengths: High diagnostic accuracy, multicenter design, external validation. Limitations: Small sample size for external validation, overfitting potential, PH diagnosis based on Echo without RHC confirmation

This table summarizes the characteristics and performance metrics of the 15 studies that either did not perform RHC, did not explicitly report its use, or replaced it with echocardiography alone for diagnostic confirmation, which we acknowledge as a methodological limitation. These studies are presented separately because the absence of invasive confirmation represents a methodological limitation that may affect diagnostic ground truth. Columns include “Author (year)” (lead author and publication year), “PH group” (pulmonary hypertension subtype studied), “Study group” (study focus), “Study design” (retrospective or prospective), “Sample size,” “Key findings,” “Outcome measures,” “Prognostic model used,” “Model type” (ML or DL), “Diagnosis method,” “Validation method,” and “Strengths and limitations,” summarizing key methodological aspects and performance outcomes

### Diagnostic and classification models

#### Model performance and validation

Reported model performance varied according to study objective, input modality and algorithmic approach. For example, Imai et al. [14] developed a DL model based on CXR images, achieving an AUC of 0.988 with a sensitivity of 0.93 and specificity of 0.98, outperforming experienced physicians in detecting PAH [14]. Similarly, DuBrock et al. [33] demonstrated that an ECG-based CNN could predict PH up to five years before clinical diagnosis (AUC 0.92 at diagnosis, remaining ≥0.80 up to 18 months pre-diagnosis) across two independent cohorts, highlighting the potential of AI for early, non-invasive screening and disease detection [72]. AUC values ranged from 0.71 to 1.00 across both ML and DL models [32, 44, 45, 62]. Diagnostic model performance varied substantially across input domains. CXR and CT-based models generally achieved moderate AUCs (for example, CXR: 0.71 in Kusunose et al. 2020/2022 [44, 45]; CT for CTEPH detection: 0.81 in Zhao et al. 2024 [64]), whereas the best-performing CXR algorithms reached very high accuracy (Imai 2024 0.988 [14]; Zou 2020 0.970/0.967 internal/external [65]). ECG-based models consistently performed in the high range, typically 0.86–0.92 (Kwon 2020 0.859/0.902 [46]; DuBrock 2024 0.92/0.88 [33], with predictive ability up to five years before diagnosis). Echocardiography-based ML also showed strong discrimination (Liao 2023 0.945/0.950 internal/external [69]). Claims/EHR-based approaches yielded high to very high AUCs (Ong 2020 0.88 [52]; Kogan 2023 0.92 [18]). Biomarker and omics studies reported exceptionally high AUCs in smaller, homogeneous cohorts (Duo 2022 0.948/0.945 [34]; Zeng 2021 AUC = 1.00 [62]), although their generalizability remains limited. Finally, multimodal models that integrated imaging and clinical data (for example, Zhao et al. 2025 [71]) achieved consistently high performance, likely reflecting the richer feature space (see Tables 1, 2).

External validation was performed in 20 studies (37.7%), partially in combination with internal validation using a held-out test set. Cross-validation was the most commonly applied strategy, frequently supplemented by a separate internal test split.

Calibration metrics and decision curve analyses were rarely reported across studies.

### Risk of bias and applicability

Risk of bias and applicability were assessed using the PROBAST+AI tool. For each domain, both methodological quality and clinical applicability were independently rated as low, moderate, or high. Based on this assessment, 44 studies (83.0%) were classified as having a low overall risk of bias. However, moderate applicability concerns were frequently identified. These were mainly related to non-representative patient populations, insufficient detail on predictor definitions and measurement, non-guideline-conforming diagnostic criteria for PH (including inconsistent use of RHC), and limited generalizability of imaging-based models to broader clinical settings.

In 15 studies (28.3%) [15–17, 39, 40, 43, 44, 46, 48, 55, 61, 62, 64, 65, 67], RHC, the diagnostic gold standard for PH, was either not performed, not explicitly reported, or replaced by echocardiography alone for diagnostic confirmation. This raised applicability concerns regarding the validity and consistency of case definitions across these studies. Subtype attribution in accordance with ESC/ERS guidelines and the Nice classification [1] was clearly reported in 38 studies (71.2%), while the remaining studies used heterogeneous PH definitions, partly without clear diagnostic specification or consistent delineation according to guideline-based criteria [1]. As RHC is not routinely performed in all patients with imaging signs of right heart strain in clinical practice, these studies nonetheless provide valuable insights into clinical and echocardiography-based AI applications in suspected PH. For completeness of the review’s evidence base, these studies were retained but are now presented separately in Table 2 to maintain a clear distinction between reference standards in the main analysis. A detailed study-level assessment of bias and applicability is provided in Supplementary Table S3.

### Prognostic and predictive models

Among the studies reviewed, nine focused on prognostic modeling, specifically aimed at predicting outcomes such as mortality [25, 31, 32, 35, 41, 57, 58, 66, 68]. While these studies leveraged imaging data and clinical endpoints, they were rarely externally validated or prospectively tested.

Alabed et al. (2022) applied a cardiac MRI-based multilinear principal component analysis (MPCA) approach to identify prognostic features across the cardiac cycle, improving 1-year mortality prediction in PAH compared with the REVEAL score (c-index 0.76 vs. 0.71) while maintaining interpretability through visualization of high-risk myocardial regions [25]. Kheyfets et al. (2023) developed a random forest model in PAH, integrating clinical, hemodynamic, and biomarker data, and achieved excellent internal (AUC 0.94) and robust external validation (AUC 0.81) for 4-year survival prediction, illustrating the potential of explainable, individualized AI-based risk assessment [68].

Prognostic models demonstrated moderate to high discriminatory ability, depending on input modality and outcome definition. Early CMR-based motion models performed at the lower bound (Dawes 2017, AUC 0.73 [66]), while registry-based Bayesian networks showed intermediate accuracy (Kanwar 2020, AUC 0.80; external 0.74–0.80 [41]). Imaging-rich or multimodal approaches achieved higher performance, with the CMR-based MPCA model by Alabed 2022 improving 1-year mortality prediction in PAH (c-index 0.83 vs. 0.71 REVEAL) [25], and the random-forest model by Kheyfets 2023 reaching AUCs of 0.94 (internal) and 0.81 (external) [68]. Further prognostic applications, such as AI-quantified fibrosis in CT (Dwivedi 2024, c-index 0.76 [35]) or multimodal EHR-based survival prediction (Suvon 2023, AUC 0.89 [58]), also demonstrated strong predictive accuracy. These results collectively highlight that greater data richness and more precise labeling enhance prognostic power (see Table 1).

## Discussion

Despite growing enthusiasm for AI in PH research, the clinical translation of ML and DL models remains limited. The observed variation in reported AUCs reflects the heterogeneity of data sources and study objectives. CXR- and CT-based models generally achieved moderate accuracy, ECG-based algorithms showed consistently higher performance, and multimodal or MRI-based prognostic models achieved the highest results, albeit often in smaller, more homogeneous cohorts. These differences likely stem from variations in data richness, label quality (RHC-confirmed vs. surrogate definitions), and cohort heterogeneity, underscoring the need for standardized endpoints, transparent reporting, and robust external validation in future studies (see Tables 1, 2).

To our knowledge, this is the first systematic review to provide a structured quality assessment of 53 studies addressing non-invasive diagnosis, phenotypic classification, and prognostication in PH. By synthesizing heterogeneous approaches using the SWiM framework and evaluating methodological rigor via the PROBAST+AI tool, we identified key limitations that currently hinder clinical implementation.

### Study cohorts and diagnosis methodology

A key limitation identified across many studies is the lack of clear differentiation between PH subgroups, particularly with respect to the current ESC/ERS classification [1]. While studies such as Swinnen et al. (2023) explicitly aimed to distinguish PAH from post-capillary PH due to left heart disease (PH-LHD) [60], this distinction was not rigorously addressed in other studies, despite its clinical importance. Differentiating between PH Groups 1 to 5 is essential, as these entities differ markedly in pathophysiology, therapeutic implications, and clinical outcomes [1]. The omission of this distinction in a considerable number of studies underscores a persistent gap between clinical priorities and prevailing practices in AI research, thereby limiting the utility of ML and DL models that do not account for the multifaceted nature of PH.

Regarding diagnostic methodology, most studies employed RHC [1]. When performed in experienced centers, RHC offers high diagnostic specificity and precision with an acceptable risk profile [8, 73]. Across 15 studies, RHC was not performed, not clearly reported, or substituted by echocardiographic assessment [15–17, 39, 40, 43, 44, 46, 48, 55, 61, 62, 64, 65, 67]. Despite its wider availability, echocardiography is insufficient for definitive diagnosis according to guideline-based algorithms [1]. To account for this methodological heterogeneity, studies that did not explicitly report RHC confirmation or used echocardiography alone for diagnosis were analyzed separately (Table 2). Although the absence of invasive confirmation limits diagnostic ground truth, these studies remain relevant as they reflect real-world clinical practice, where RHC is not routinely performed in all patients with imaging signs of right heart strain. Moreover, they provide complementary insights into the development and validation of non-invasive AI models for screening or triage applications. Nevertheless, the use of inconsistent diagnostic standards introduces potential bias, limits comparability, and hinders both model performance and clinical translation. In addition, the decision to perform RHC itself may represent a classification factor, as patient selection for invasive confirmation often differs across PH subgroups and disease stages.

### Model performance: machine learning vs. deep learning

The studies included in this review predominantly employed supervised ML models such as support vector machines (SVM), random forests, and gradient boosting machines (GBM) for structured clinical data. These models demonstrated promising discriminatory performance, with AUC values ranging from 0.73 to 1.00 [62, 66]. However, most were developed for binary classification tasks, such as distinguishing PH from healthy controls or identifying at-risk individuals. While such classifiers may support initial screening, they fall short of addressing dynamic clinical needs, including the prediction of long-term outcomes, therapeutic response, or continuous hemodynamic parameters. The limited adoption of regression-based ML approaches limits the clinical applicability of current models for longitudinal monitoring and individualized prognostic assessment.

In contrast, DL approaches, primarily based on CNNs, were mainly applied to imaging data. A considerable number of studies focused specifically on CXR analysis [14, 15, 43–45, 48, 65]. These studies demonstrated the feasibility of using DL for non-invasive detection of PH and PAH, with reported AUC values ranging from 0.71 to 1.00 [32, 44, 45]. The use of widely available CXR data underscores the potential of DL models for scalable, non-invasive screening in PH. However, several limitations remain. The opaque nature of CNN-based models challenges clinical acceptance [74, 75]. None of the included studies appear to have employed explainable AI (XAI) techniques such as saliency maps, Gradient-weighted Class Activation Mapping (Grad-CAM), or layer-wise relevance propagation.

### Prognostic models and long-term outcomes

Although several studies have explored AI-based prognostic modeling in pulmonary hypertension, most remain limited by small sample sizes, lack of external validation, and insufficient reporting of model interpretability. Many models did not adequately quantify the relevance of input features, and clinical transparency was often insufficiently addressed. This lack of interpretability hampers clinical applicability, as clinicians require explainable and actionable outputs to inform patient management and therapeutic decisions [76].

Despite these challenges, AI-driven prognostic tools hold considerable promise for advancing PH management by enabling earlier risk stratification and personalized treatment strategies [11].

### Data heterogeneity and multi-modal integration

A major limitation across the studies was the heterogeneity of data sources. The studies included in this review utilized various combinations of data types. This diversity complicates direct comparisons between studies and the identification of reproducible predictors. Notably, only a small number of studies integrated multiple data modalities, such as imaging and clinical data, to potentially enhance predictive performance [17, 58, 71]. Only seven studies incorporated advanced data types such as proteomics, transcriptomics, lipidomics, or radiomics [29, 30, 34, 36, 53, 54, 70].

### Validation methods and generalizability

A critical limitation across the studies was the insufficient attention to model validation. While most studies relied solely on internal cross-validation, only a limited number employed independent hold-out sets or external datasets. Notably, Zhao et al. (2025) and Bordag et al. (2023) highlighted the value of external validation, demonstrating robust model performance across distinct cohorts and datasets [30, 64]. However, the lack of consistent external validation across the studies included in this review raises concerns about the generalizability of the proposed ML models. Small sample sizes, particularly from single-center cohorts, increase the risk of overfitting and limit broader applicability. Moreover, none of the studies fully met the methodological and reporting standards assessed using the PROBAST+AI tool [23], reflecting persistent gaps in model transparency, reproducibility, and bias control. While several approaches demonstrate considerable innovation, many remain at an early proof-of-concept stage. Robust external validation and prospective multicenter testing are essential to address these concerns [77, 78].

### Model interpretability and ethical considerations

A key barrier to the clinical adoption of AI models is their limited interpretability and transparency. While metrics such as AUC and accuracy are important indicators of model performance, ML and DL applications in healthcare must also be comprehensible to clinicians and transparent in their decision-making logic. Techniques from the field of XAI, including SHapley Additive exPlanations (SHAP) and Local Interpretable Model-agnostic Explanations (LIME), are critical for fostering trust and enabling the meaningful integration of model outputs into clinical workflows [79–81].

In addition to technical concerns, ethical challenges such as data privacy, algorithmic fairness, and bias against underrepresented populations are often insufficiently addressed. Such bias may arise from imbalanced datasets, unrepresentative training populations, or opaque model development processes. These challenges require collaborative strategies, including representative data selection and transparent model auditing [82]. A comprehensive taxonomy of bias sources and fairness strategies highlights the persistent risk of discriminatory outcomes if fairness is not explicitly addressed throughout the AI development process [83]. Addressing these issues is essential for the responsible and equitable implementation of AI in clinical care.

Trust in AI is not established by validation metrics alone but rather emerges throughout the development process. Winter and Carusi (2022) demonstrated that validation and trust are co-constructed iteratively through continuous interaction between algorithm developers and clinical users. Their study on AI-assisted early diagnosis of PH emphasized how crucial steps such as data curation, label refinement, and the choice of benchmarks are shaped collaboratively, often through tacit, practice-oriented input that is not captured in formal reporting [84]. In this sense, validation is not a static technical endpoint but an evolving process embedded in clinical workflows. Acknowledging and integrating these collaborative dynamics may be essential for developing AI systems that are robust, interpretable, and clinically acceptable.

## Summary

This systematic review analyzed 53 studies applying ML and DL to PH, focusing on non-invasive models for diagnosis, classification, and prognostication. Key aspects included model inputs, algorithm types, validation strategies, and subgroup differentiation. While ML- and DL-based approaches demonstrated promising accuracy, limited external validation, methodological heterogeneity, and the common failure to address subgroup-specific analyses continue to constrain clinical applicability.

### Strengths and limitations

This systematic review provides a comprehensive and methodologically rigorous synthesis of current ML and DL applications in PH research. One key strength lies in the structured evaluation of studies based on clinical intent, model design, and phenotypic focus, which allows for a differentiated assessment of algorithmic potential across the PH spectrum. Additional strengths include the prospective registration in PROSPERO (CRD420251074202) [22], the consistent application of transparent inclusion criteria, and adherence to PRISMA methodology [21]. Study quality and reporting were critically appraised using the recently updated PROBAST+AI tool [23], specifically developed for assessing ML-based prediction models. The synthesis and reporting were also guided by the SWiM guideline, which supports transparent evidence presentation in the absence of formal meta-analysis due to methodological heterogeneity [24]. Another strength is the review’s focus on PH subgroup differentiation, which addresses a clinically important but often overlooked aspect in PH research.

Several limitations should be acknowledged. First, the number of eligible studies remains limited, reflecting the early stage of AI application in this field. Second, substantial heterogeneity in input features, outcome definitions, and performance metrics limited comparability. Third, the lack of access to source code, model parameters, or detailed preprocessing steps in most studies hindered transparency and reproducibility, which impacts the robustness of the review’s findings.

### Future directions

To advance the clinical utility of AI in PH, future research should prioritize phenotypically precise model development across all subgroups defined by the current classification [1]. Substantial differences in pathophysiology, therapeutic response, and prognosis between PAH and other forms of PH necessitate subgroup-specific algorithms trained and validated on clearly stratified patient populations. Rigorous model validation must become standard practice, including not only internal cross-validation and independent hold-out testing, but also external validation to ensure broader applicability. Given the relative rarity of PH and its subtypes, collaborative multicenter registries and federated learning approaches may help overcome current limitations in sample size and data diversity. To increase model transparency and foster clinician trust, explainability techniques such as SHAP values, attention mechanisms, or class activation mapping (CAM) should be routinely implemented and clearly reported. By mitigating the black-box nature of AI models, these tools enhance clinical interpretability and help identify biologically plausible predictors, similar to the feature selection process in methods like Least Absolute Shrinkage and Selection Operator (LASSO). Furthermore, integrating structured clinical and imaging data with unstructured modalities such as free-text reports or waveform data holds promise for improving model performance and robustness. Clinical implementation of AI models in PH should complement rather than replace established clinical workflows, with particular attention to interoperability with EHRs and prospective validation. In addition, future research should aim to systematically consider health economic implications, for example by evaluating whether AI-based tools can contribute to more efficient diagnostic pathways or resource allocation. Close collaboration between clinicians, data scientists, health economists, software engineers, and regulatory bodies is essential to ensure that future AI applications meet the standards of safety, transparency, clinical and health economic relevance required for real-world adoption.

## Conclusions

AI holds considerable promise to support earlier diagnosis, individualized risk assessment, and data-informed therapeutic decision-making in PH. Current ML and DL models show encouraging performance in diagnostic and prognostic applications based on non-invasive clinical and imaging data. However, progress toward clinical translation remains limited by small sample sizes, single-center designs, methodological heterogeneity, and the lack of external validation and standardized subgroup phenotyping aligned with current ESC/ERS guidelines. Future research should prioritize harmonized development and reporting practices, transparent diagnostic labeling, and robust multicenter validation to enable safe and effective integration of AI tools into clinical care.

## Questions for future research

- How can AI models in PH be trained on data strictly aligned with current clinical and hemodynamic definitions?
- What methodological approaches can enhance the generalizability of AI tools across PH subgroups and clinical environments?
- Can explainable AI increase transparency and foster clinical acceptance in PH applications?
- How can AI support early risk stratification and individualized therapy guidance, especially in PAH?
- How can AI tools in PH be prospectively validated through real-world, multicenter study designs?

## Supplementary Information

Additional file 1.

Additional file 2.

Additional file 3.

## Data Availability

This systematic review is based on publicly available data from previously published studies. As no original data were collected or generated, no new datasets are available. All relevant data from the included studies are cited in the manuscript and summarized in the main text and supplementary tables. The corresponding review protocol was prospectively registered and is publicly available in PROSPERO (registration number: CRD420251074202). No analytic code was generated, as data synthesis was conducted narratively following the SWiM (Synthesis Without Meta-analysis) approach. A pre-defined data extraction form was used but is not publicly available; it can be obtained upon reasonable request from the corresponding author. Further inquiries regarding specific studies or data can also be directed to the corresponding author.

## Abbreviations

AI: Artificial intelligence
Ao: Aorta
AUC: Area under the curve
AUROC: Area under the receiver operating characteristic curve
AUPRC: Area under the precision recall curve
BERT: Bidirectional encoder representations from transformers
BNP: Brain natriuretic peptide
CatBoost: Categorical boosting
CMR: Cardiovascular magnetic resonance
CNN: Convolutional neural network
CT: Computed tomography
CTEPH: Chronic thromboembolic pulmonary hypertension
CTPA: CT pulmonary angiography
CXR: Chest X-ray
DAFIT: Data-augmented feature integration technique
DL: Deep learning
Echo: Echocardiography
ECG: Electrocardiogram
EHR: Electronic health record
F1-Score: Harmonic mean of precision and recall
GSE: Gene expression omnibus series
HR: Hazard ratio
ICD-9/10: International classification of diseases, ninth/tenth revision
IL-2: Interleukin-2
IL-9: Interleukin-9
LASSO: Least absolute shrinkage and selection operator
LV: Left ventricle
MAE: Mean absolute error
ML: Machine learning
MLP: Multilayer perceptron
MPCA: Multilinear principal component analysis
MRI: Magnetic resonance imaging
mPAP: Mean pulmonary arterial pressure
MSKCC: Memorial Sloan Kettering Cancer Center
NPV: Negative predictive value
PA: Pulmonary artery
PAH: Pulmonary arterial hypertension
PAP: Pulmonary arterial pressure
PASP: Pulmonary arterial systolic pressure
PH: Pulmonary hypertension
PH-LD: Pulmonary hypertension due to lung disease
PH-LHD: Pulmonary hypertension due to left heart disease
PIOPED: Prospective investigation of pulmonary embolism diagnosis
PPG: Photoplethysmography
PPV: Positive predictive value
PRISMA: Preferred reporting items for systematic reviews and meta-analyses
PROBAST+AI: Prediction model risk of bias assessment tool for artificial intelligence
R^2^: Coefficient of determination
RHC: Right heart catheterization
ROC: Receiver operating characteristic
RV: Right ventricle
RVEF: Right ventricular ejection fraction
SVM: Support vector machine
sPAP: Systolic pulmonary arterial pressure
SVR: Support vector regression
TAN: Tree-augmented naïve Bayes
TPR: True positive rate
TRPG: Tricuspid regurgitant pressure gradient
TRV: Tricuspid regurgitation velocity
VUMC: Vanderbilt University Medical Center
WGCNA: Weighted gene co-expression network analysis
XGBoost: Extreme gradient boosting
