# Supplementary material for: Artificial intelligence in pulmonary hypertension: a systematic review
Source: Eur J Med Res. 2025 Dec 8;30:1225. doi: 10.1186/s40001-025-03557-5 (PMC12690818; doi:10.1186/s40001-025-03557-5)
Supplement: Supplementary file 1 — Additional file 1. [file 40001_2025_3557_MOESM1_ESM.docx]

**Supplementary Table S1. Search Strategy**

Detailed search strategy for the systematic review of artificial-intelligence applications in pulmonary hypertension. The table outlines search strings, Boolean logic, and applied filters for each database. MEDLINE (via PubMed) and Google Scholar were searched using identical search logic with syntax adjustments for Google Scholar, which also indexes full-text information. Reference lists of all electronically identified studies and relevant review articles were screened manually, yielding six additional studies. The full PubMed query is provided for reproducibility. The search strategy was iteratively refined by adjusting keyword groupings and Boolean logic to verify that previously known relevant publications were consistently retrieved by the final search strategy.

| **Database / Source** | **Fields searched** | **Boolean Logic and Operators** | **Limits / Filters Applied** | **Notes** |
| --- | --- | --- | --- | --- |
| **MEDLINE (via PubMed)** | Titles, Abstracts, and MeSH terms | Boolean operators **AND** and **OR** used to combine related MeSH and free-text terms; parentheses applied to group concepts | Publication years 2016–2025; Humans; English language | MeSH indexing used to identify relevant subject headings |
| **Google Scholar** | Titles, Abstracts, and Full-text content (indexed automatically) | Boolean operators **AND** and **OR** applied as in PubMed; minor syntax adjustments for Google Scholar | Publication years 2016–2025; English language | Used as a complementary source to identify additional relevant studies, as Google Scholar also indexes full-text information |
| **Manual reference screening** | Reference lists of all electronically identified and relevant articles were manually screened to identify additional eligible studies | — | Same inclusion limits as database search | Six additional studies identified |

**Search String**("Pulmonary Hypertension"[MeSH] OR "pulmonary hypertension"[tiab] OR "pulmonary arterial hypertension"[tiab])
AND ("Artificial Intelligence"[MeSH] OR "artificial intelligence"[tiab] OR "machine learning"[tiab] OR "deep learning"[tiab])
AND ("diagnosis"[tiab] OR "prediction"[tiab] OR "prognosis"[tiab] OR "phenotyping"[tiab] OR "classification"[tiab] OR "risk stratification"[tiab] OR "non-invasive"[tiab] OR "survival"[tiab] OR "mortality"[tiab] OR "electrocardiography"[tiab] OR "echocardiography"[tiab] OR "chest X-ray"[tiab] OR "computed tomography"[tiab] OR "magnetic resonance imaging"[tiab] OR "electronic health records"[tiab])
