# Supplementary material for: Artificial intelligence in pulmonary hypertension: a systematic review
Source: Eur J Med Res. 2025 Dec 8;30:1225. doi: 10.1186/s40001-025-03557-5 (PMC12690818; doi:10.1186/s40001-025-03557-5)
Supplement: Supplementary file 3 [file 40001_2025_3557_MOESM3_ESM.odt]

Supplementary Table 2. PROBAST+AI Assessment Criteria

This supplementary table summarizes the criteria used to assess risk of bias and applicability in ML and DL studies related to PH. The evaluation follows the PROBAST+AI framework, which covers four key domains: participants, predictors, outcomes, and analysis. Each domain includes specific signaling questions to evaluate methodological quality, reproducibility, and clinical relevance. The final two columns provide overall judgments for each study: “Overall Quality” reflects the overall risk of bias, while “Overall Applicability” summarizes the generalizability of the study findings to real-world clinical settings. This structured assessment aims to improve transparency and support evidence-based implementation of ML and DL in healthcare.

| Study | Participants & Data Sources (Quality) | Participants & Data Sources (Applicability) | Predictors (Quality) | Predictors (Applicability) | Outcomes (Quality) | Outcomes (Applicability) | Analysis (Quality) | Analysis (Applicability) | Overall Quality | Overall Applicability |
| --- | --- | --- | --- | --- | --- | --- | --- | --- | --- | --- |
| Alabed et al. (2022) | High | Moderate | High | High | High | High | High | High | Low risk of bias | Moderate concern |
| Anand et al. (2024) | High | High | High | High | High | High | High | High | Low risk of bias | Moderate concern |
| Aras et al. (2023) | Medium | High | High | Medium | High | Medium | Medium | Medium | Medium risk of bias | High  concern |
| Argiento et al. (2024) | High | High | High | High | High | High | High | High | Low risk of bias | Moderate concern |
| Bauer et al. (2021) | High | Moderate | High | High | High | High | High | High | Low risk of bias | Moderate concern |
| Bordag et al. (2023) | Medium | Medium | High | Medium | High | Medium | High | Medium | Low risk of bias | Moderate concern |
| Chettrit et al. (2019) | Medium | Medium | High | Medium | Medium | Medium | High | Medium | Low risk of bias | Moderate concern |
| Dawes et al. (2017) | Medium | Medium | High | High | High | High | High | High | Low risk of bias | Moderate concern |
| Diller et al. (2022) | Medium | Medium | High | High | High | High | High | Medium | Low risk of bias | Moderate concern |
| DuBrock et al. (2024) | Medium | Medium | High | High | Medium | Medium | High | Medium | Low risk of bias | Moderate concern |
| Duo et al. (2022) | Medium | Medium | High | Medium | High | Medium | High | Medium | Low risk of bias | Moderate concern |
| Dwivedi et al. (2024) | High | Medium | High | High | High | High | High | High | Low risk of bias | Low   concern |
| Errington et al. (2023) | Medium | Medium | High | High | High | Medium | High | Medium | Low risk of bias | Moderate concern |
| Fortmeier et al. (2022) | Medium | High | High | High | High | Medium | High | Medium | Low risk of bias | Moderate concern |
| Guo et al. (2025) | Medium | Medium | High | Medium | High | High | High | Medium | Low risk of bias | Moderate concern |
| Gwalitza et al. (2024) | High | Medium | High | Medium | High | High | High | Medium | Low risk of bias | Moderate concern |
| Han et al. (2024) | Medium | Medium | High | Medium | High | High | High | Medium | Low risk of bias | Moderate concern |
| Hu et al. (2023) | High | Medium | High | Medium | High | High | High | Medium | Low risk of bias | Moderate concern |
| Hyde et al. (2023) | Medium | Medium | High | High | High | Medium | High | Medium | Low risk of bias | Moderate concern |
| Imai et al. (2024) | High | Medium | High | Medium | High | High | High | Medium | Low risk of bias | Moderate concern |
| Kanwar et al. (2020) | High | High | High | Medium | High | High | High | Medium | Low risk of bias | Moderate concern |
| Kheyfets et al. (2023) | High | Medium | High | High | High | High | High | High | Low risk of bias | Low   concern |
| Kiely et al. (2019) | High | High | High | Medium | High | High | High | Medium | Low risk of bias | Moderate concern |
| Kishikawa et al. (2025) | High | Medium | High | High | High | Medium | High | Medium | Low risk of bias | Moderate concern |
| Kıvrak et al. (2023) | High | Medium | High | High | High | High | High | Medium | Low risk of bias | Moderate concern |
| Kogan et al. (2023) | Medium | Medium | High | Medium | High | Medium | High | Medium | Medium risk of bias | Moderate concern |
| Kusunose et al. (2022) | High | High | High | Medium | High | High | High | Medium | Low risk of bias | Moderate concern |
| Kusunose et al. (2020) | High | Medium | High | High | High | High | High | Medium | Low risk of bias | Moderate concern |
| Kwon et al. (2020) | Medium | Medium | High | Medium | High | Medium | Medium | Medium | Medium risk of bias | Moderate concern |
| Leha et al. (2019) | High | Medium | High | High | High | High | High | Medium | Low risk of bias | Low   concern |
| Liao et al. (2023) | High | Medium | High | Medium | High | High | High | Medium | Low risk of bias | Moderate concern |
| Liu et al. (2024) | Medium | Medium | Medium | Medium | Medium | Medium | Medium | Medium | High risk of bias | High  concern |
| Liu et al. (2022) | Medium | Medium | Medium | Medium | Medium | Medium | Medium | Medium | High risk of bias | High  concern |
| Lungu et al. (2016) | High | High | High | Medium | High | High | High | Medium | Low risk of bias | Moderate concern |
| Matsunaga et al. (2024) | High | Medium | High | Medium | High | High | High | Medium | Low risk of bias | Moderate concern |
| Murayama et al. (2024) | High | Medium | High | Medium | High | High | High | Medium | Low risk of bias | Moderate concern |
| Nemati et al. (2024) | High | High | High | Medium | High | High | High | Medium | Low risk of bias | Moderate concern |
| Ong et al. (2020) | High | Medium | High | High | High | High | High | Medium | Low risk of bias | Moderate concern |
| Priya et al. (2021) | High | High | High | Medium | High | High | High | Medium | Low risk of bias | Moderate concern |
| Priya et al. (2021) | High | Medium | High | Medium | High | High | High | Medium | Low risk of bias | Moderate concern |
| Ragnarsdottir et al. (2024) | High | High | High | Medium | High | High | High | Medium | Low risk of bias | Moderate concern |
| Schuler et al. (2022) | High | Medium | High | Medium | High | High | High | Medium | Low risk of bias | Moderate concern |
| Shikhare et al. (2022) | High | High | High | Medium | High | High | High | Medium | Low risk of bias | Moderate concern |
| Suvon et al. (2023) | High | Medium | High | High | High | High | High | Medium | Low risk of bias | Moderate concern |
| Sweatt et al. (2019) | High | Medium | High | Medium | High | High | High | Medium | Low risk of bias | Moderate concern |
| Swift et al. (2021) | High | Medium | High | Medium | High | High | High | Medium | Low risk of bias | Moderate concern |
| Swinnen et al. (2023) | High | Medium | High | Medium | High | High | High | Medium | Low risk of bias | Moderate concern |
| Yang et al. (2024) | High | Medium | Medium | Medium | High | Medium | Medium | Medium | High risk of bias | High  concern |
| Zeng et al. (2021) | High | Medium | High | Medium | High | Medium | Medium | Medium | Medium risk of bias | Moderate concern |
| Zhang et al. (2023) | High | Medium | Medium | Medium | High | High | High | Medium | Low risk of bias | Moderate concern |
| Zhao et al. (2024) | High | Medium | High | Medium | High | High | High | Medium | Low risk of bias | Moderate concern |
| Zhao et al. (2025) | High | Medium | Medium | Medium | High | Medium | High | Medium | Medium risk of bias | Moderate concern |
| Zou et al. (2020) | High | Low | High | Medium | High | Medium | High | Medium | Medium risk of bias | High  concern |
